# Diet-induced obesity reduces bone marrow T and B cells and promotes tumor progression in a transplantable Vk*MYC model of multiple myeloma

**DOI:** 10.1038/s41598-024-54193-8

**Published:** 2024-02-13

**Authors:** Tonje Marie Vikene Nedal, Siv Helen Moen, Ingrid Aass Roseth, Synne Stokke Tryggestad, Kristin Roseth Aass, Gunhild Garmo Hov, Hanne Hella, Anne-Marit Sponaas, Therese Standal

**Affiliations:** 1 Department of Clinical and Molecular Medicine, Centre of Molecular Inflammation Research, Faculty of Medicine and Health Sciences, Norwegian University of Science and Technology (NTNU), Trondheim, Norway; 2 Department of Research, Nord-Trøndelag Hospital Trust, Levanger, Norway; 3 Department of Clinical Chemistry, St. Olavs Hospital, Trondheim, Norway; 4 Department of Hematology, St. Olavs Hospital, Trondheim, Norway

**Keywords:** Cancer, Immunology

## Abstract

Obesity is associated with an increased risk of developing multiple myeloma (MM). The molecular mechanisms causing this association is complex and incompletely understood. Whether obesity affects bone marrow immune cell composition in multiple myeloma is not characterized. Here, we examined the effect of diet-induced obesity on bone marrow immune cell composition and tumor growth in a Vk*MYC (Vk12653) transplant model of multiple myeloma. We find that diet-induced obesity promoted tumor growth in the bone marrow and spleen and reduced the relative number of T and B cells in the bone marrow. Our results suggest that obesity may reduce MM immune surveillance and thus may contribute to increased risk of developing MM.

## Introduction

Multiple myeloma (MM) is a cancer originating from plasma cells within the bone marrow. It is the second most common hematological cancer, affecting mainly elderly people with a median age at diagnosis of about 70 years^1^. In addition to high age, obesity is among the risk factors. Epidemiological studies have shown that obese persons have a higher chance of developing MM and MGUS^2–4^ and a higher chance of progressing from MGUS to MM^5,6^. Moreover, excess body weight during childhood and early adulthood is associated with increased risk of developing MM later in life^7^.

That obesity may drive MM disease establishment or disease progression is also supported by experimental models. For example, it has been shown that diet-induced obesity (DIO) enabled establishment of 5TGM1 myeloma cells in C57Bl/6J mice^8^, a mouse strain that is not normally permissive for growth of these cells. Others, using different clones of the Vk*MYC cells, found that DIO increased the number tumor cells in the spleen^9,10^ and in the bone marrow^10^. It has been shown that increased bone marrow adiposity, that is often correlated with obesity, may lead to increased expression of pro-inflammatory cytokines and adipokines, which may enhance myeloma cell survival or proliferation^8,10,11^. The reasons for the increased risk of developing MM in obese humans or mice are however incompletely understood and whether obesity affects bone marrow immune cell composition in myeloma is not characterized.

In this study we examined the effect of DIO on bone marrow immune cell composition and tumor growth in the Vk*MYC (Vk12653) myeloma mouse model^12^. We find, in accordance with previous studies, that DIO leads to increased tumor load in the spleen and the bone marrow. Importantly, we also find that DIO significantly reduces number of T cells and B cells in the bone marrow. Our result suggest that obesity may not only provide a pro-survival effect on the MM cells but may reduce cancer immune surveillance and thereby increased risk of developing MM.

## Results

### Diet-induced obesity promoted tumor growth in bone marrow and spleen

**Figure 1:**
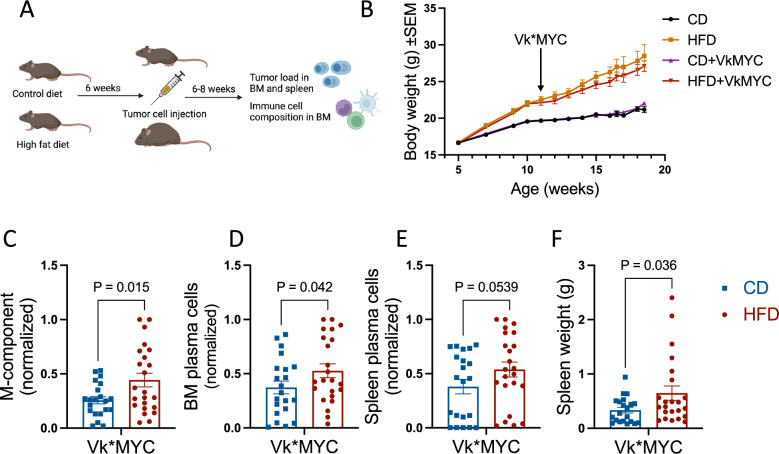
DIO promotes tumor growth in BM and spleen. (**A**) Overview of the experimental setup. Endpoint were 6 or 8 weeks after VK12653 tumor cell injection. (**B**) Body weight of mice measured from change of diets until end of experiment (N = 16–23 mice per group). (**C**) Serum M-component (normalized gamma globulin/albumin ratio) at endpoint. (**D**) Normalized percent plasma cells (CD138 + B220-/live cells) in the BM at endpoint. (**E**) Normalized percent plasma cells (CD138 + B220- /live cells) in the spleen at endpoint. (**F**) Spleen weight at endpoint. Statistical significance was determined by one-tailed T-test. Normalization (**C**–**E**) was done using the min–max normalization formula, as described in the “Methods” section.

To investigate the effect of obesity on tumor cell growth and immune cell composition we fed five weeks old C57BL6/J mice a control diet (CD) or a calorie-rich high fat diet (HFD) for six weeks before i.v. injection of Vk12653 myeloma cells^12^ (Fig. 1A). Mice on HFD gained significantly increased body weight compared with mice on the control diet (Fig. 1B). They also had significantly increased amount of abdominal fat at endpoint as assessed by μCT scanning of a few mice in each diet group (N = 4) (Supplementary Fig. S1A).

At end point mice with diet-induced obesity (DIO) had a higher tumor load measured as circulating levels of M-component (Fig. 1C) and percent plasma cells in the bone marrow compared with control mice (Fig. 1D). Further, percent plasma cells in the spleens (Fig. 1E) as well as spleen weight (Fig. 1F) were increased in obese tumor mice compared with control tumor mice. Spleen weight correlated with percent tumor cells in the spleen (Supplementary Fig. S1B). Taken together, these results support that DIO promoted tumor growth in BM and spleen.

### Diet-induced obesity leads to a reduction in T and B cells in the bone marrow

**Figure 2:**
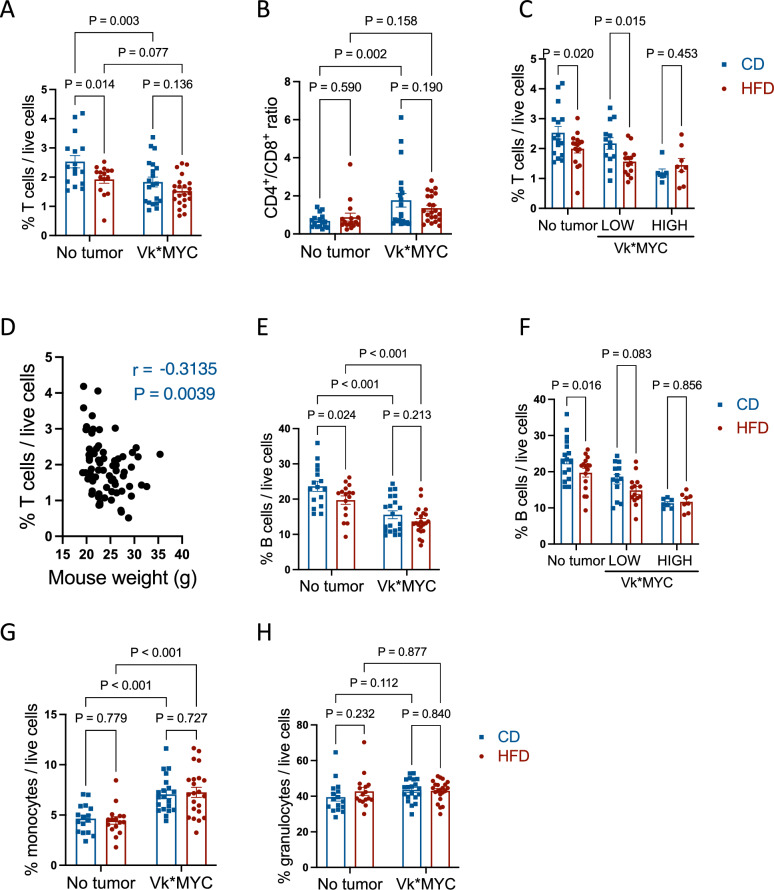
DIO reduces number of lymphocytes in the BM. Right femur and tibia were flushed of BM at endpoint and stained as described in the “Methods” section. (**A**) Percent T cells (CD3^+^CD19^−^/live cells). (**B**) Ratio of CD4^+^ T cells/ CD8^+^ T cells. (**C**) Percent T cells (CD3^+^CD19^−^/live cells) in non-tumor mice and tumor-harboring mice grouped according to low (< 10% PC in BM) or high (> 10% PC in BM) tumor load. (**D**) Correlation between mouse weight and percent T cells (CD3^+^CD19^−^/live cells) in the bone marrow. r = Spearman correlation coefficient. (**E**) Percent B cells (CD19^+^CD3^−^/live cells). (**F**) Percent B cells (CD19^+^CD3^−^/live cells) in non-tumor mice and tumor mice grouped according to low (< 10% PC in BM) or high (> 10% PC in BM) tumor load. (**G**) Percent monocytes (CD11b^+^Ly6C^+^LY6G^−^/live cells). (**H**) Percent granulocytes (CD11b^+^Ly6C^−^/Ly6G^+^/live cells). Statistical significances (P < 0.05) were determined by two-way ANOVA followed by Fischer’s LSD.

To address whether DIO influenced immune cell composition in the BM we identified T cells (CD3^+^/CD19^−^), B cells (CD19^+^/CD3^−^), monocytes (CD11b^+^/Ly6C^+^/Ly6G^−^) and granulocytes (Cd11b^+^/Ly6C^−^/Ly6G^+^) by flow cytometry of BM cells flushed from hind legs (see Supplementary Fig. S2 for gating strategy). We found that the T cell population was decreased in HFD-fed mice compared with mice on the control diet (Fig. 2A). As expected, this was also the case when tumor-harboring mice were compared with healthy mice (Fig. 2A). Furthermore, there was a negative correlation between tumor load in the BM and percent T cells (Supplementary Fig. S3A). The DIO-induced reduction in T cells affected both CD8^+^ and CD4^+^ cells since the CD4/CD8 ratio did not change depending on diet. On the other hand, compared with no-tumor control mice, presence of tumor led to increased CD4/CD8 ratio, suggestive of a more specific tumor-induced depletion of CD8T cells (Fig. 2B). There was no significant diet-induced difference in T cells in tumor-bearing mice (Fig. 2A). However, when we divided tumor-harboring mice into groups based on having low (< 10%) or high (≥ 10%) levels of plasma cells in the BM (Supplementary Fig. S3B) we found that DIO led to significantly reduced BM T cells in the group of mice with low BM tumor load (Fig. 2C). We also observed a significant negative correlation between percent T cells in the BM and mouse weight, further supporting that obesity leads to a reduction of T cells in the BM (Fig. 2D). Taken together, these data support that obesity negatively impact BM T cells in healthy mice and in mice with low BM tumor load. When tumor load is high, T cell numbers are severely reduced, and the diet-induced reduction in BM T cells has less impact.

Similar to what was observed for the T cells, B cells in the BM were significantly reduced in obese mice compared with lean mice, and in tumor-harboring mice compared with control mice irrespectively of diet (Fig. 2E). There was also a significant negative correlation between tumor load in BM and percent B cells (Supplementary Fig. S3C). The effect of DIO on B cells was not significant in tumor-harboring mice (Fig. 2E) and when looking into the effect of DIO in groups of mice with low or high tumor load, there was no significant change in B cells in either group (Fig. 2F). Thus, both DIO and tumor leads to a reduction of B cells in the BM, but the reduction of B cells as a result of DIO is not significant in tumor-harboring mice.

DIO did not influence percent T and B cells in the spleen per se, however tumor-harboring mice on a HFD had a reduction in spleen B cells compared with mice on the CD (Supplementary Fig. S4A,B). Moreover, tumor-harboring mice had significantly reduced percent B and T cells in the spleen compared with non-tumor mice irrespective of diet (Supplementary Fig. S4A,B). These data suggest that a DIO-reduction of T and B lymphocytes primarily affects the bone marrow.

The diet had little impact on the relative number of BM cells of the myeloid lineage. The monocyte population (CD11b^+^Ly6C^+^Ly6G^−^) was increased in tumor-mice on either diet compared with mice without tumors but did not differ between obese mice and mice on the control diet (Fig. 2G). In contrast, relative number of granulocytes (CD11b + Ly6C-Ly6G +) was similar across all groups, suggesting that neither diet nor presence of tumor significantly alter number of granulocytes in the BM in this model (Fig. 2H). In summary, tumor increased the relative number of monocytes in the bone marrow, but levels of these cells in the BM were not affected by diet-induced obesity per se.

## Discussion

The main finding of this study is that DIO led to increased tumor burden and reduced numbers of T and B lymphocytes in the BM. A proper immune response is critical for MM disease control^13,14^ and a reduction in BM lymphocytes upon obesity may thus have consequences for tumor establishment.

Tumor-specific T cells are present in the bone marrow of MGUS and MM patients^15–18^, and presence of tumor-recognizing T cells reduces the likelihood for progression from MGUS to MM^19^, supporting that T cells at least in the initial phase of disease can control tumor growth. Moreover, an efficient T cell response characterize long-term survival^20,21^, and clonal T cell expansions has been associated with lower tumor burden and survival advantage^22,23^. The Vk*MYC model replicates the human disease in many ways, including the role of T cells for tumor control^24,25^. Although not investigated in the current study, we hypothesize that the observed reduction in BM T cells may have contributed to the increase in tumor load. The effect of DIO on immune cells was most evident in mice without tumors and when tumor burden was low, while the diet did not significantly impact frequency of T cells when tumor burden was high. We hypothesize that in mice with a high tumor load the effect of tumor cells on the BM microenvironment is dominating and may mask the effect of obesity. Another possibility is that tumor-derived factors may have counteracted the effect of the DIO on lymphocytes. Importantly, while tumor mice had a higher CD4/CD8 ratio compared with non-tumor mice, suggesting a specific reduction of CD8 cells due to the presence of tumor, this was not the effect of DIO, which caused a general reduction in both CD4 and CD8 BM T cells.

We found that the diet increased visceral adiposity but we did not evaluate how the diet affected adiposity in the bone marrow and spleen. Thus, it may be that the difference in effect of DIO on lymphocytes is related to a different effect on adiposity in the two organs. Moreover, while the spleen acts as a reservoir of lymphocytes the generation and replenishment of the lymphoid lineage takes place in the BM. Indeed, a high- fat diet has previously been shown to disrupt the HSC niche due to increased numbers of adipocytes at the expense of osteoblasts, resulting in reduced lymphopoiesis^26^.

DIO promoted tumor growth in the spleen, despite that there was no change in T cell frequency in this organ. This support that DIO also have a direct effect on the tumor cells, for example mediated by increased circulating levels of tumor- promoting hormones and cytokines such as. e.g. leptin, IGF-1, IL-6 or angiotensin-II, which has previously been shown to be increased upon obesity and that may act as myeloma promoting factors^8,10,27^.

The importance of a proper B cell response in the initial phases of tumor development is not well characterized, although a decline in BM B cells has been observed in MM patients^28^. How a potential reduction in B cells upon obesity may influence tumor progression is therefore not so easy to predict. Importantly, our data is in line with others that have also reported a reduction in bone marrow T and B cells in obese C57/Bl6 mice^26,29,30^.

Obesity has been associated with an exhausted/senescent T cell phenotype^31,32^, which also characterizes the T cells in MM^14^. Whether T cell dysfunction is a greater problem in obese versus lean patients or arises earlier in disease development in obese myeloma patients compared with lean patients should be explored in future studies. Moreover, we did not include markers for NK or NKT cells, and the effect of obesity on these important immune cell subsets is at present unknown. Interestingly, leptin has been shown to counteract the anticancer effect of iNKT cells in the 5T33 MM model^27^.

In conclusion, we have shown that DIO leads to a reduction in T and B cells in the BM and more aggressive tumor growth. The effect of obesity on BM T-and B cell repertoire in patients with MGUS and smoldering myeloma should be determined as our data indicate that obesity may reduce BM tumor immune surveillance.

## Materials and methods

### In vivo experiments

Female C57BL/6J mice were provided by Janvier (France) at the age of four weeks. Upon arrival at the specific pathogen free unit at the Comparative Medicine Core Facility (CoMed, NTNU, Trondheim, Norway), the mice were acclimatized for one week. Five mice were housed per cage. Mice were randomly allocated to the different study groups. At five weeks of age the mice were given either a control diet (CD.88137, 13% fat, 15.7 MJ/kg metabolizable energy (ME)) or a high fat/western diet (TD.88137, 42% fat, 19.1 MJ/kg ME) (ssniff, Germany). Detailed information of the diet can be found in Supplementary Table S1. The mice were given fresh food every week. Mice were weighed every other week for the first month and then once every week for the rest of the study. A score sheet was used to monitor the animal welfare in relation to defined human endpoints.

After six weeks on the diet, mice were injected with either Vk12653 cells (50,000 cells) or PBS intravenously in the tail, alternating between CD and HFD cages (Fig. 1A). The Vk12653 cells were generously provided by Marta Chesi, Mayo Clinic^12^. Blood was collected by bleeding from the saphenous vein four weeks after tumor injection, and every other week for the duration of the experiment. At endpoint, blood was collected by cardiac puncture of mice anesthetized by isoflurane followed by euthanasia by cervical dislocation. Blood was spun at 2000 G for 10 min at RT to collect serum. Both hind legs and the spleen were collected at endpoint to isolate cells for flow cytometric analyses. Mice from two separate experiments were combined. After excluding mice without tumor take (i.e. no visible peak in serum protein electrophoresis, N = 10) the groups were as follows: Vk*MYC on CD (N = 21) and Vk*MYC on HFD (N = 23). Control mice without tumors: CD (N = 16) and HFD (N = 16). The experiment was not blinded.

### Ethical approval

All experiments in animals were performed in compliance with the ARRIVE guidelines (https://arriveguidelines.org) and the relevant guidelines and regulations of. Animal handling and procedures were approved by the Norwegian Food Safety Authority (FOTS ID 25087).

### Antibodies

Anti-Mouse CD138 BV421 (281-2), CD19 PeCy7 (6D5), B220 Alexa647/CD45R(RA3-6B2), CD4 FITC (GKI.1), CD8 AF700 (53-6.7), CD3PE (17A2), CD11b FITC (M1/70), Ly-6C PerCP (HK1.4), Ly-6G APC (1A8) and CD16/CD32 (93) were from BioLegend (SanDiego, CA, USA).

### M-component measurement

Serum M-components were measured using the urine procedure for protein electrophoresis on CAPILLARYS 2 (Sebia). The urine procedure was used due to low sample volume. Serum samples and controls were diluted 1:80 with dialysis buffer before analysis. One serum control sample were analyzed in each series. M-components are reported as ratio of gamma globulin/albumin.

### Flow cytometry

Bone marrow cells from the right hind legs were flushed by centrifugation of cleaned bones at 10,000*g* for 15 s. Spleen cells were isolated by crushing the spleen in a culture dish containing 10 ml 5% FCS/RPMI followed by passing of the cell suspension through a 70 µm cell strainer. A proportion of spleen cells were frozen for later analysis. Freshly isolated cells from bone marrow and spleen were treated with RBC Lysis Buffer (Invitrogen, Waltham, Massachusetts, USA) before staining. All cells were stained with anti-mouse CD16/CD32 (Fc block) on ice for 20 min before staining for the different cellular markers. Flushed bone marrow cells were stained with antibodies recognizing lymphocytes (CD138, B220, CD3, CD19, CD4, CD8) or myeloid cells (CD11b, Ly-6C and Ly-6G). Freshly obtained spleen cells were stained with anti-CD138 and anti-B220 to identify plasma cells. Spleen T cells and B cells were analyzed in frozen samples that were thawed and treated with DNAse (Roche Diagnostics GmbH, Mannheim, Germany), stained with LIVE/DEAD Fixable Aqua Dead Cell Stain, stained for CD3 and CD19 and fixed with fixation buffer (BioLegend, SanDiego, CA, USA) before flow cytometric analyses. Flow cytometry was performed using LSR II (BD Biosciences, San Jose, CA, USA) and FACS Diva software (BD Bioscience) and data were analyzed with FlowJo v10.7.2 (TreeStar, Ashland, OR, USA). For gating strategies, see Supplementary Fig. S1.

### Statistical analysis

Statistical analyses were performed using GraphPad Prism 10 for MacOS. Comparisons between two groups were performed by student T test and when comparing more than two groups by two-way ANOVA. Correlations between two parameters were evaluated by calculating Spearman correlation coefficients. To enable statistical analyses on tumor load from two separate experiments, tumor data (M component, percent plasma cells in BM, percent plasma cells in spleen) were normalized. Normalization was done using the min–max normalization formula, spreading the values from each experiment between 0 and 1. Min–max normalization formula: z_i_ = (xi − min(x))/(max(x) −min(x)), z_i_: the ith normalized value in the dataset, x_i_: the ith value in the dataset, min(x): the minimum value in the dataset, max(x): the maximum value in the dataset. P values < 0.05 were considered significant.

### Supplementary Information

Supplementary Figures.

Supplementary Information 2.

Supplementary Table 1.

## Data Availability

All data generated or analyzed during this study are included in this published article and its supplementary information files.
