# Supplementary material for: Diet-induced obesity reduces bone marrow T and B cells and promotes tumor progression in a transplantable Vk*MYC model of multiple myeloma
Source: Sci Rep. 2024 Feb 13;14:3643. doi: 10.1038/s41598-024-54193-8 (PMC10864380; doi:10.1038/s41598-024-54193-8)
Supplement: Supplementary file 1 — Supplementary Figures. [file 41598_2024_54193_MOESM1_ESM.pptx]

## Slide 1
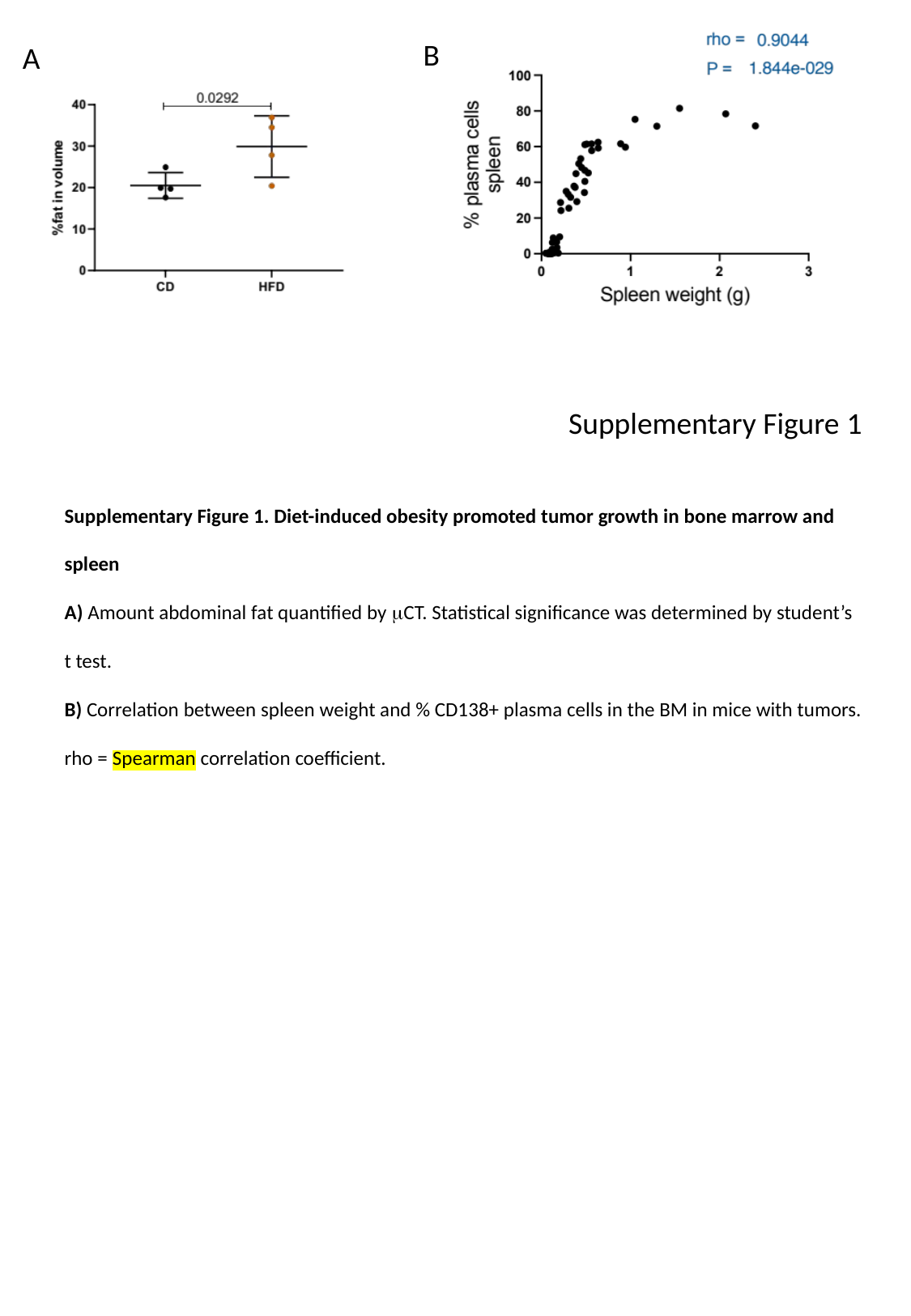

B
A
Supplementary Figure 1
Supplementary Figure 1. Diet-induced obesity promoted tumor growth in bone marrow and spleen
A) Amount abdominal fat quantified by mCT. Statistical significance was determined by student’s t test.
B) Correlation between spleen weight and % CD138+ plasma cells in the BM in mice with tumors. rho = Spearman correlation coefficient.

## Slide 2
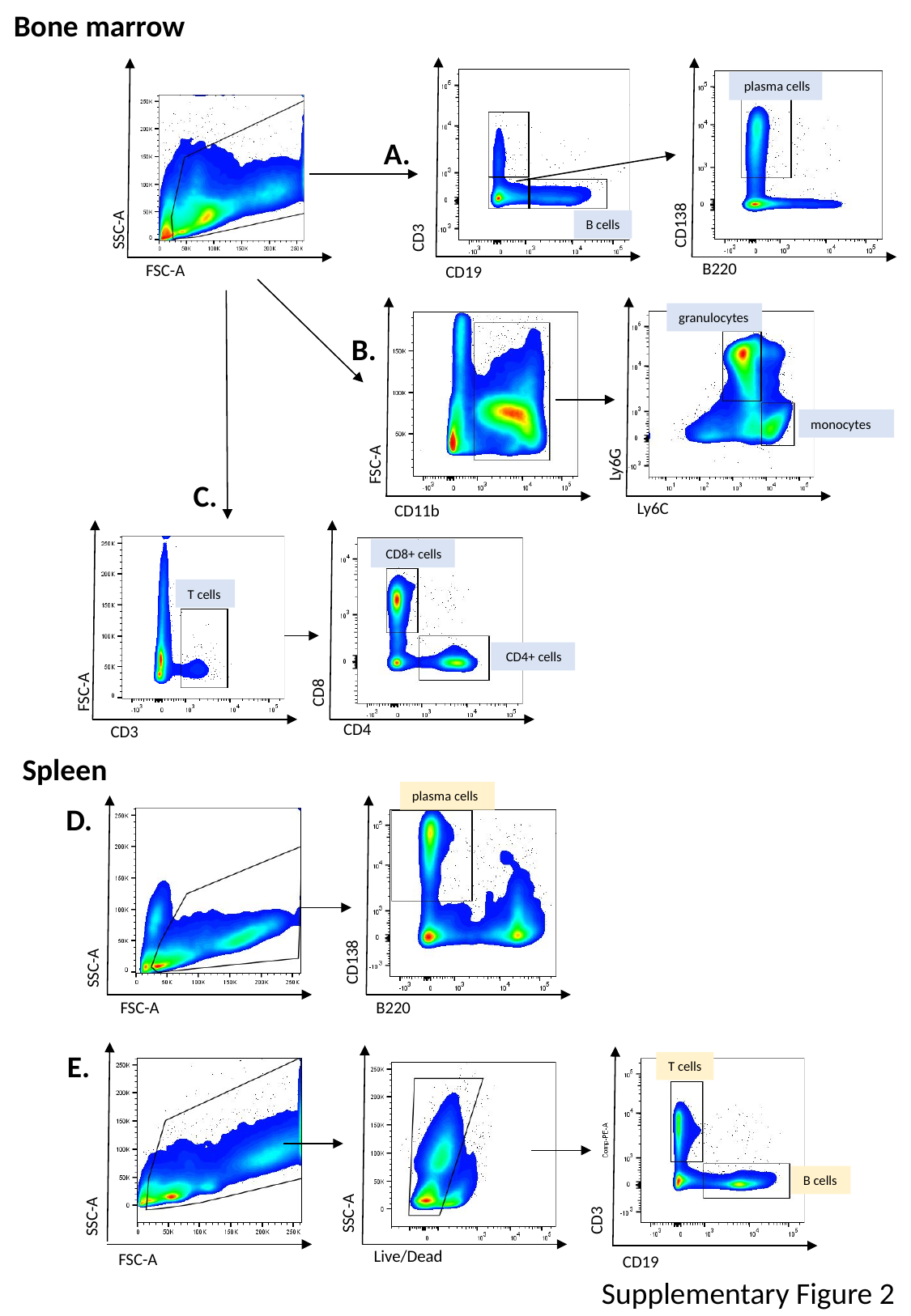

Bone marrow
 plasma cells
A.
CD138
SSC-A
B cells
CD3
B220
FSC-A
CD19
granulocytes
B.
monocytes
FSC-A
Ly6G
C.
Ly6C
CD11b
 CD8+ cells
T cells
 CD4+ cells
CD8
FSC-A
CD4
CD3
Spleen
plasma cells
D.
CD138
SSC-A
B220
FSC-A
E.
T cells
B cells
SSC-A
CD3
SSC-A
Live/Dead
FSC-A
CD19
Supplementary Figure 2

## Slide 3
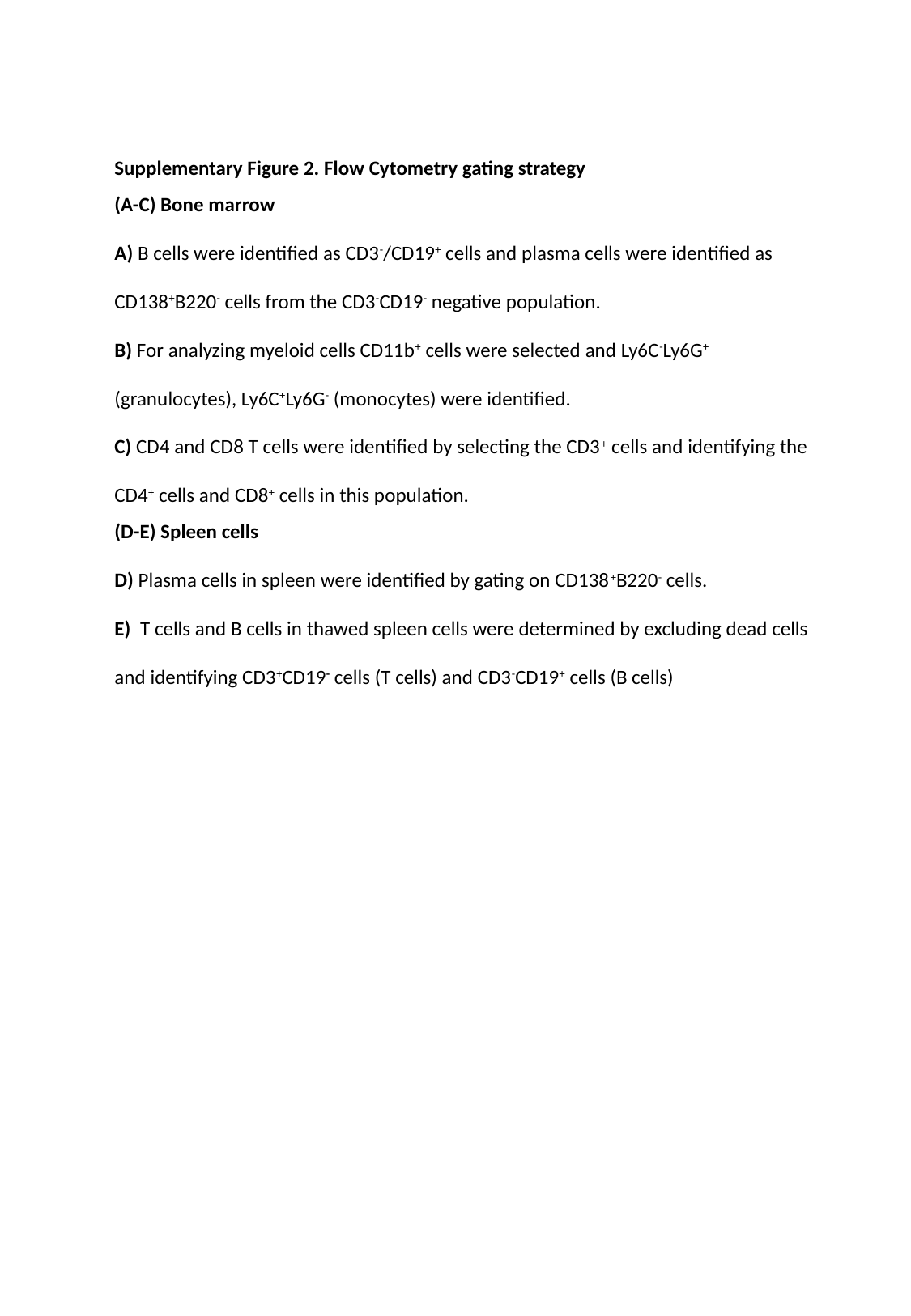

Supplementary Figure 2. Flow Cytometry gating strategy
(A-C) Bone marrow
A) B cells were identified as CD3-/CD19+ cells and plasma cells were identified as CD138+B220- cells from the CD3-CD19- negative population.
B) For analyzing myeloid cells CD11b+ cells were selected and Ly6C-Ly6G+ (granulocytes), Ly6C+Ly6G- (monocytes) were identified.
C) CD4 and CD8 T cells were identified by selecting the CD3+ cells and identifying the CD4+ cells and CD8+ cells in this population.
(D-E) Spleen cells
D) Plasma cells in spleen were identified by gating on CD138+B220- cells.
E) T cells and B cells in thawed spleen cells were determined by excluding dead cells and identifying CD3+CD19- cells (T cells) and CD3-CD19+ cells (B cells)

## Slide 4
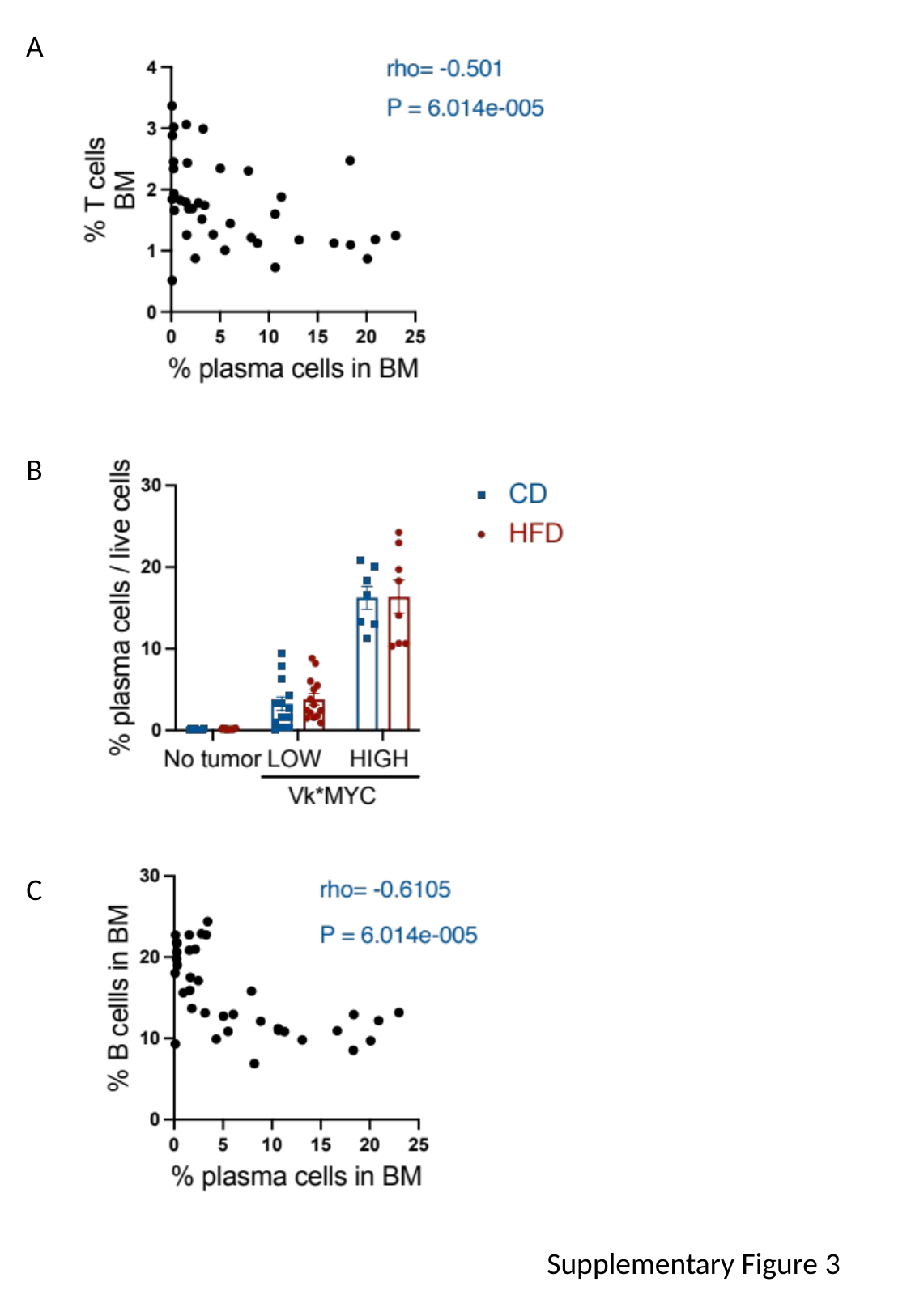

A
B
C
Supplementary Figure 3

## Slide 5
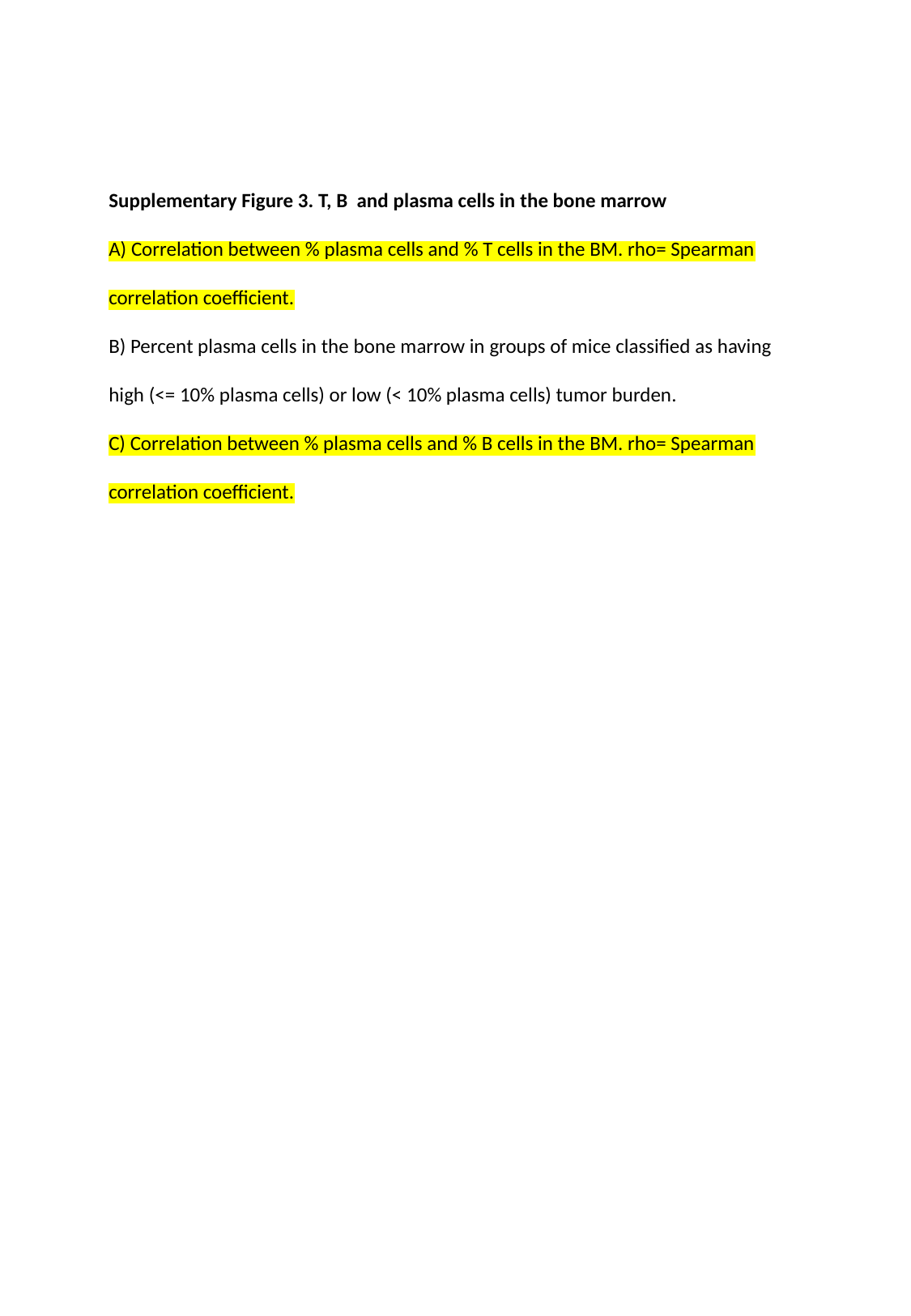

Supplementary Figure 3. T, B and plasma cells in the bone marrow
A) Correlation between % plasma cells and % T cells in the BM. rho= Spearman correlation coefficient.
B) Percent plasma cells in the bone marrow in groups of mice classified as having high (<= 10% plasma cells) or low (< 10% plasma cells) tumor burden.
C) Correlation between % plasma cells and % B cells in the BM. rho= Spearman correlation coefficient.

## Slide 6
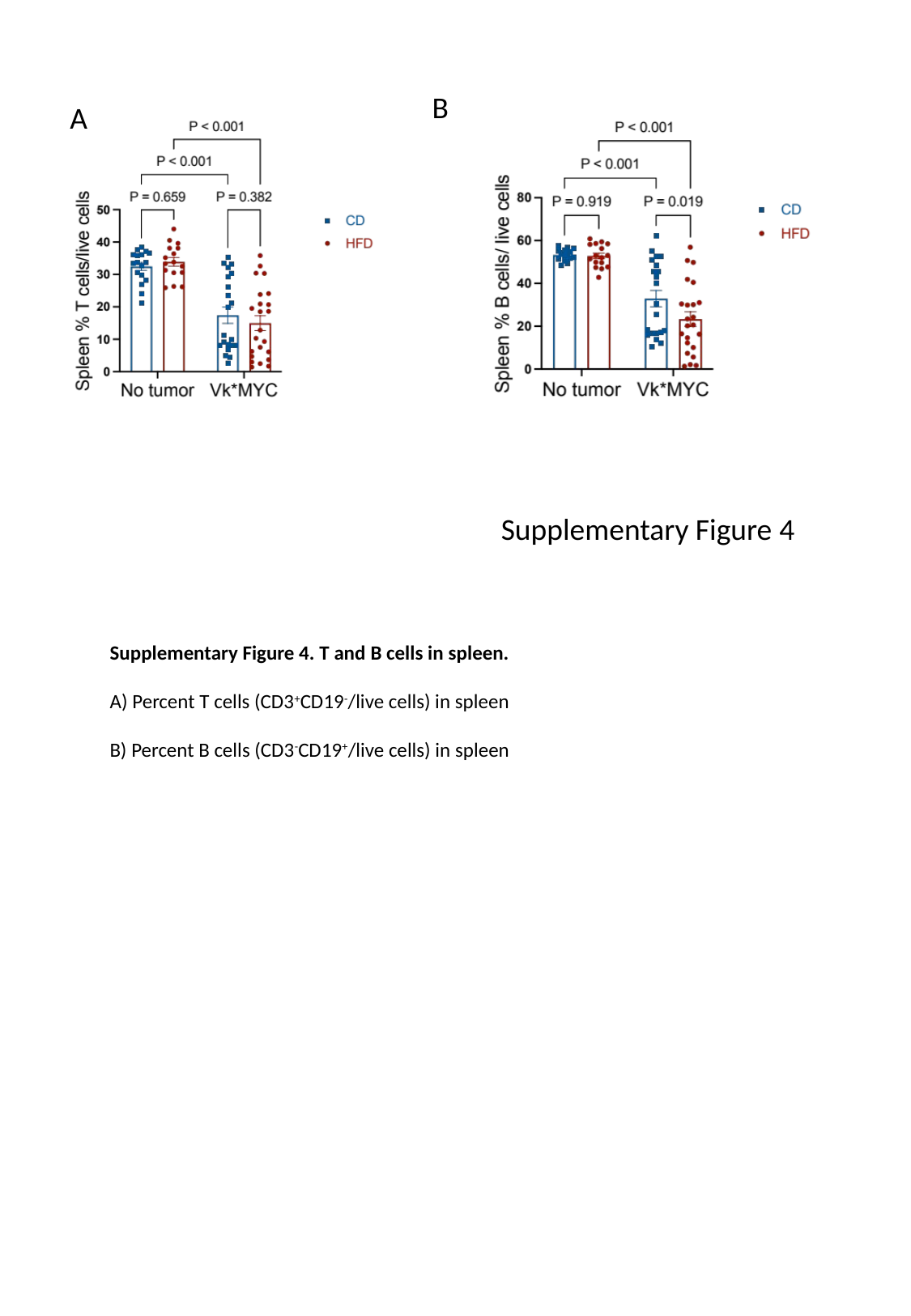

B
A
Supplementary Figure 4
Supplementary Figure 4. T and B cells in spleen.
A) Percent T cells (CD3+CD19-/live cells) in spleen
B) Percent B cells (CD3-CD19+/live cells) in spleen
