# Supplementary material for: Diet-induced obesity reduces bone marrow T and B cells and promotes tumor progression in a transplantable Vk*MYC model of multiple myeloma
Source: Sci Rep. 2024 Feb 13;14:3643. doi: 10.1038/s41598-024-54193-8 (PMC10864380; doi:10.1038/s41598-024-54193-8)
Supplement: Supplementary file 2 — Supplementary Information 2. [file 41598_2024_54193_MOESM2_ESM.docx]

# Supplementary Methods

**uCT fat analysis**

CT scanning of the abdominal region was performed on a group of mice after euthanasia using a µCt scanner (Skyscan 1176, Bruker, Kontich, Belgium). Images were acquired using the following settings: 35 um voxel resolution, 0.5 mm aluminum filter, 50 kV voltage, 500 uA current, 120 ms exposure time, rotation step of 0.5 degrees, dynamic range = 0 - 0.04, frame averaging of 3. Reconstruction of the images were done in NRecon (version 1.7.4.6) using a ring artifact correction of 8, beam hardening of 34 % and a smoothing of 1. Abdominal fat was analyzed using CT Analyzer (version 1.18.8.0) in a region of the abdomen. The region of interest was set by first measuring the length between the point where the first femur head connects with the hip (acetabulum) and the point where the sacroiliac joint ends. 60% of this length was added below the acetabulum, while 100% of this length was added above the sacroiliac joint to make up the region of interest for the fat analysis. Cross section images were made using CT Analyzer.
