# Supplementary material for: Diet-induced obesity reduces bone marrow T and B cells and promotes tumor progression in a transplantable Vk*MYC model of multiple myeloma
Source: Sci Rep. 2024 Feb 13;14:3643. doi: 10.1038/s41598-024-54193-8 (PMC10864380; doi:10.1038/s41598-024-54193-8)
Supplement: Supplementary file 3 — Supplementary Table 1. [file 41598_2024_54193_MOESM3_ESM.pdf]

Supplementary Table 1: Diet composition

	WD (TD.88137)	CD (CD.88137)
Gross energy (GE)	21.8 MJ / kg	18.3 MJ / kg
Metabolizable energy (ME)	19.1 MJ / kg	15.7 MJ / kg
Fat	42 kJ %	13 kJ %
Protein	15 kJ %	18 kJ %
Carbohydrates	43 kJ %	69 kJ %
Dietary composition:	The Western diet contains 21 % butter fat and app. 0.21 % cholesterol (0.15 % supplemented). This diet will therefore induce hyperlipidemic disorders, hypercholesterolemia and atherosclerosis, i.e. plaque formation, in ApoE- and Ldlr-deficient mice.	This diet has been designed as control diet to the Western diets. It is characterized by low sugar and fat contents; the butter fat has been exchanged by soybean oil.
**Crude nutrients (%)**		
Crude protein (N x 6.25)	17.3	17.3
Crude fat	21.1	5.1
Crude ash	4.2	3.9
Starch	14.4	38.5
N free extracts	49.8	64.7
Crude fibre	5.0	5.0
Sugar	34.3	11.0
**Minerals (%)**		
Calcium	0.76	0.76
Phosphorus	0.45	0.48
Ca/P	1.69 : 1	1.57 : 1
Sodium	0.24	0.16
Magnesium	0.10	0.10
Potassium	0.54	0.55
**Fatty Acids (%)**		
C 4:0	0.80	
C 6:0	0.53	
C 8:0	0.29	
C 10:0	0.63	
C 12:0	0.72	0
C 14:0	2.22	0.02
C 16:0	5.60	0.61
C 17:0	0.14	0.01
C 18:0	2.05	0.19
C 20:0	0.04	0.02
C 16:1	0.38	0.01
C 18:1	4.65	1.27
C 18:2	0.38	2.63
C 18:3	0.11	0.29
**Amino Acids (%)**		
Lysine	1.43	1.43
Methionine	0.93	0.73
Cystein	0.07	0.27
Met+Cys	1.00	1.00
Threonine	0.76	0.76
Tryptophan	0.22	0.22
Arginine	0.67	0.67
Histidine	0.52	0.52
Valine	1.20	1.20
Isoleucine	0.97	0.97
Leucine	1.71	1.71
Phenylalanine	0.89	0.89
Phe+Tyr	1.80	1.80
Glycine	0.34	0.34
Glutamic acid	3.88	3.88
Aspartic acid	1.28	1.28
Proline	1.97	1.97
Serine	1.03	1.03
Alanine	0.52	0.52
**Vitamins (mg/kg)**		
Vitamin A (IU)	15000	15000
Vitamin D3 (IU)	1500	1500
Vitamin E	150	150
Vitamin K (as MNB)	20	20
Thiamine (B1)	26	26
Riboflavin (B2)	16	16
Pyridoxine (B6)	16	16
Cobalamin (B12) (ug)	30	30
Nicotinic acid	49	49
Pantotheic acid	55	55
Folic acid	16	16
Biotin (ug)	300	300
Choline	920	920
**Trace elements (mg/kg)**		
Iron	49	49
Manganese	22	22
Zinc	41	41
Copper	11	11
Iodine	0.3	0.3
Selenium	0.2	0.2
**Cholesterol (mg/kg)**	2.070	0
Cholesterol (%)	0.21	0
